# Real-World Journey of Unresectable Stage III NSCLC Patients: Current Dilemmas for Disease Staging and Treatment

**DOI:** 10.3390/jcm11061738

**Published:** 2022-03-21

**Authors:** Abed Agbarya, Walid Shalata, Alfredo Addeo, Andriani Charpidou, Kristof Cuppens, Odd Terje Brustugun, Mirjana Rajer, Marco Jakopovic, Mihai V. Marinca, Adam Pluzanski, Jeroen Hiltermann, António Araújo

**Affiliations:** 1 Oncology Department, Bnai Zion Medical Center, Haifa 3339419, Israel; 2 The Legacy Heritage Oncology and Larry Norton Institute, Soroka Medical Center and Ben Gurion University, Beer Sheva 84105, Israel; walid_sh@clalit.org.il; 3 Oncology Department, University Hospital of Geneva, 1205 Geneva, Switzerland; alfredo.addeo@hcuge.ch; 4 “Sotiria” General Hospital, 11527 Athens, Greece; dcharpidou@yahoo.gr; 5 Department of Pulmonology and Thoracic Oncology, Jessa Hospital, 3500 Hasselt, Belgium; kristof.cuppens@jessazh.be; 6 Section of Oncology, Drammen Hospital Vestre Viken Hospital Trust, 3004 Drammen, Norway; ot.brustugun@gmail.com; 7 Institute of Oncology, 1000 Ljubljana, Slovenia; mrajer@onko-i.si; 8 Clinical Center for Pulmonary Diseases Jordanovac, University Hospital Centre Zagreb, 10000 Zagreb, Croatia; marko.jakopovic@kbc-zagreb.hr; 9 Medical Oncology (IRO Iasi), Grigore T. Popa University of Medicine & Pharmacy, 700115 Iasi, Romania; m.marinca@gmail.com; 10 Maria Sklodowska-Curie National Research Institute of Oncology, 00-001 Warsaw, Poland; adam.pluzanski@pib-nio.pl; 11 Department of Pulmonary Diseases, University Medical Center Groningen, University of Groningen, 9712 CP Groningen, The Netherlands; t.j.n.hiltermann@umcg.nl; 12 Department of Medical Oncology, Centro Hospitalar Universitário do Porto, 4099-001 Porto, Portugal; antonio.araujo@chporto.min-saude.pt; 13 School of Medicine and Biomedical Sciences Abel Salazar—ICBAS, 4050-313 Porto, Portugal

**Keywords:** lung neoplasms, immunotherapy, practice patterns, health care surveys

## Abstract

Daily-practice challenges in oncology have been intensified by the approval of immune checkpoint inhibitors (ICI). We aimed to outline current therapy policies and management of locally advanced unresectable stage III non-small-cell lung cancer (NSCLC) in different countries. One thoracic oncologist from each of the following countries—Belgium, Croatia, Greece, Israel, the Netherlands, Norway, Poland, Portugal, Romania, Slovenia, and Switzerland—participated in an electronic survey. Descriptive statistics were conducted with categorical variables reported as frequencies and continuous variables as median and interquartile range (IQR) (StataSE-v15). EBUS (endobronchial ultrasound bronchoscopy) was used either upfront or for N2 confirmation. Resectability is still a source of disagreement; thus, decisions vary within each multidisciplinary team. Overall, 66% of stage III patients [IQR 60–75] undergo chemoradiation therapy (CRT); concurrent CRT (cCRT) accounts for most cases (~70%). Performance status is universally used for cCRT eligibility. Induction chemotherapy is fairly weighted based on radiotherapy (RT) availability. Mean time to evaluation after RT completion is less than a month; ICI consolidation is started within six weeks. Durvamulab expenditures are reimbursed in all countries, yet some limiting criteria exist (PD-L1 ≥ 1%, cCRT). No clear guidance on therapies at Durvamulab progression exist; experts agree that it depends on progression timing. Given the high heterogeneity in real-world practices, standardized evidence-based decisions and healthcare provision in NSCLC are needed.

## 1. Introduction

Lung cancer remains the leading cause of cancer incidence and mortality worldwide, with 1 reported death every 18 s [1,2]. In 2020, more than 2 million newly diagnosed cases and 1.8 million deaths were disclosed, accounting for 18% of total cancer deaths [3]. Non-small-cell lung cancer (NSCLC) represents the most common lung tumor (80–85% of cases) [4,5] of which approximately 1/3 are already locally advanced at diagnosis, with around 15–17% being unresectable at presentation [6,7,8].

Unresectable stage III locoregionally or locally advanced NSCLC comprises a highly heterogenous group of clinical conditions with regard to patient fitness, primary tumor size and distribution, resulting in a wide range of prognosis and therapeutic alternatives [6,7,8]. Although international guidelines for the management of these tumors indicate the use of a physical exam, biopsy, and imaging tests—including positron emission tomography (PET), thoracic computed tomography and/or real-time endobronchial ultrasound (EBUS) approaches, challenges for stage III NSCLC diagnosis and staging are still prevalent in clinical practice, especially regarding procedures’ timing. Additionally, no single definition of ‘resectability’ at this advanced stage is universally accepted, and the treatment approach is usually determined on a case-by-case basis by a multidisciplinary team of experts comprising medical and radiation oncologists, pulmonologists, thoracic surgeons, medical imaging and ideally a pathologist and molecular biologist [9].

Over the past decades, the standard treatment for unresectable locally advanced NSCLC has been definitive chemoradiation therapy (CRT). Previous meta-analysis showed the superiority of an integrated approach compared to radiotherapy (RT) alone. In particular, platinum-based regimens combined with RT provided a 13% proportional reduction in the annual risk of death for these patients, giving a 5-year absolute benefit in the overall survival of 2.2% compared to RT [10,11]. Concurrent chemoradiation therapy (cCRT) has also demonstrated a greater gain compared to sequential treatment (sCRT), with an overall survival increase of 4.5% at 5 years [12]. However, despite numerous treatment improvement attempts, most patients rapidly progress after cCRT—with nearly 40% experiencing locoregional recurrence, and approximately 50% or more developing distant metastasis. Only 15–25% of patients are alive at 5 years after diagnosis, percentages that have remained relatively unchanged over time [4,13]. Two strategies aiming at improving patients’ outcomes have been developed in the last few years: the induction chemotherapy (ChT) before cCRT and the consolidation therapy, which is defined as treatment administered after the culmination of a defined number of ChT cycles with or without RT. Yet, it is still unclear whether these treatments can significantly improve patients’ outcomes [14,15].

The recent introduction of immune checkpoint inhibitors (ICIs) has significantly modified the therapeutic algorithm at this stage of the disease [16,17]. Durvamulab, a high-affinity human immunoglobulin G1 monoclonal antibody that blocks the binding of PD-L1 on tumor cells or antigen-presenting cells with PD-1 and CD80, was used as a consolidation therapy after/following cCRT, with the aim of improving the curative outcomes of cCRT treatment. Results from the preplanned interim analysis of the PACIFIC trial [18] demonstrated that Durvamulab is associated with benefit in progression-free survival (PFS) as compared with placebo, with median values of 16.8 months and 5.6 months, respectively [stratified hazard ratio (HR) for disease progression or death, 0.52; 95% CI, 0.42–0.65; *p* < 0.001]. In 2018, with a median follow-up of 25.2 months, the first overall survival (OS) interim analysis was performed, revealing a statistically significant and clinically meaningful increase in this outcome (stratified HR for death, 0.68; 99.73% CI, 0.47 to 0.997; *p* = 0.0025). Benefit in PFS and OS was observed regardless of the stage (IIIA and IIIB), histology (non-squamous and squamous histology) and PD-L1 status. In a post-hoc analysis, presented at the 2018 Congress of the European Society for Medical Oncology (ESMO), patients with a PD-L1 score ≥1% treated with Durvamulab had a significantly longer median PFS compared with placebo [17.8 vs. 5.6 months, respectively, HR (95% CI): 0.46 (0.33–0.64)] and prolonged median OS (not reached vs. 29.1 months, HR (95% CI): 0.53 (0.36–0.77)], after the same median follow-up of 25.2 months (range 0.2–43.1). Recent long-term follow-up analyses showed that clinical benefits of Durvamulab are sustained and consistent with the primary results, with a median OS after 5 years of 47.5 months vs. 29.1 months for placebo within the intention-to-treat (ITT) population [HR (95% CI): 0.72 (0.59–0.89)] and 61.3 months vs. 29.6 months for the PD-L1 ≥ 1% group [HR (95% CI): 0.61 (0.44–0.85)]. Studies also confirm a favorable safety profile of Durvamulab, with manageable adverse events and tolerability, which is consistent with other ICI [18,19,20,21,22,23]. Based on this achievement, several regulatory agencies in the US, Canada, Australia, Japan, Malaysia, Singapore, India, the United Arab Emirates, and the European Union approved Durvamulab as a standard of care for this setting [17].

Nonetheless, the integration of Durvamulab in clinical practice underscores the need for multidisciplinary and uniform decision-making processes, which raise new dilemmas and challenges, as well as changes in work routines, which are faced daily by lung cancer specialists. Several questions regarding patients’ journey still need to be clarified, including disease diagnosis and staging, identification of biomarkers, patients’ re-evaluation process, timing of ICI, and eligibility of patients who will benefit most from this therapy. Additionally, as the efficacy and safety of Durvamulab in populations that were not previously included in the PACIFIC trial are still unknown (e.g., multiple comorbidities, poor performance status etc.), therapeutic approaches can vary widely. This in turn widens the gap between real-world practices among different countries and intensifies the discussion on therapies’ access and reimbursement [24,25].

The present study aimed to characterize, by means of a clinical specialists’ panel, the current treatment policies and management of locally advanced unresectable stage III NSCLC in different countries that have approved the usage of Durvamulab.

## 2. Materials and Methods

This study was a survey of expert opinions among clinical specialists from 11 countries. We initially approached leading thoracic oncologists working in a referral center from 16 European countries, and 11 physicians agreed to participate in this study. The study was based on an electronic survey (Sep/2020) followed by several virtual meetings to discuss locally advanced unresectable stage III NSCLC and the current challenges with regards to: disease staging, resectability, CRT eligibility, consolidation with immunotherapy, and treatment upon progression. The questions addressed several issues based on a personal participant approach, local societies’ guidelines (if they exist) and country approval status. The questionnaire was developed especially for this study and reviewed by experts in the field. Participants took an average time of 10–12 min to complete the survey.

Procedures followed the standards for scientific research and were performed according to the Declaration of Helsinki. Physicians were fully informed regarding the nature of the study, the procedures for data recording, and the voluntary nature of their participation.

Data were systematically collected in Excel spreadsheets. Descriptive statistics were performed with categorical variables reported as frequencies and continuous variables as median and interquartile range (IQR) (StataSE v15).

## 3. Results

Physicians from Belgium, Croatia, Greece, Israel, the Netherlands, Norway, Poland, Portugal, Romania, Slovenia, and Switzerland agreed to participate in the study and met the inclusion criteria.

Table 1 depicts the current practices for stage III NSCLC staging. All thoracic oncologists consider the PET scan to be a standard imaging procedure to be performed either at diagnosis on all patients (*n* = 7/11) or only on candidates for surgery or radical CRT (*n* = 4/11). Most clinicians also perform a brain MRI as part of the clinical staging (*n* = 8/11). Regarding pathological staging, all practitioners attested to using EBUS; however, some performed EBUS upfront (*n* = 6/11) while others only for N2 confirmation (*n* = 5/11). PD-L1 and EGFR/ALK assessments are conducted for stage III patients in most countries (9/11 and 7/11, respectively). Additional biomarker assessment (i.e., molecular profiling) was mentioned by eight experts. Next-Generation Sequencing (NGS) is routinely used in Belgium, Portugal, and Switzerland; in Greece and in Israel, this profile is only performed privately (i.e., paid for by the patient or their insurer). ROS-1 is evaluated in Croatia, Greece, Norway, and Slovenia, while Greece and Slovenia also employ a BRAF assessment; in Slovenia additional profiling of KRAS and NTRK is a common practice.

According to all 11 physicians, treatment decisions for unresectable stage III NSCLC patients are always grounded in multidisciplinary teams’ discussions (Table 2). Resectability differs among countries, and decisions vary within the different multidisciplinary teams. Nonetheless, regarding unresectable patients that are the focus of this manuscript, 7/11 physicians stated that there are no differences in their treatment decision between stages IIIA and IIIB, while the remaining 4/11 agree that the substage impacts their approach. The percentage of unresectable patients in stage III is also highly variable among nations (ranging from 60% to 90%) with a median of 70% [IQR 60–80]. Neoadjuvant ChT alone is used by most experts (*n* = 8/11), especially for potentially operable cases.

The exact definition of cCRT was not unanimous. While all physicians agreed that it broadly refers to the simultaneous use of ChT and RT, the number of concomitant cycles varied between one and two; some physicians define cCRT only when ChT and RT are started simultaneously, at D1 of cycle 1 (see Table 2).

Experts estimated that a median of 66% of stage III patients [IQR 60–75] undergo CRT, where cCRT accounts for 66% [IQR 63–75] and the complement of sCRT accounts for the remaining 34% [IQR 25–38]. Performance status is a unanimous cCRT eligibility criterion. Additional parameters included patients’ age, comorbidities, access to therapy, and/or tumor size. Lung function testing is regarded by most oncologists as a comorbidity approach when qualifying for cCRT. Cardiology and pulmonology were the most frequently mentioned consultations to qualify patients for cCRT. All physicians recommend CRT for patients who arbored molecular aberrations at diagnosis.

Induction ChT is considered mainly when RT is not immediately available (*n* = 5/11). ChT is usually platinum combined with etoposide; however, protocols vary widely among settings. All of the experts recommended between 60–66 Gy using intensity modulated RT; around half also use 3D-CRT (see Table 2). Among unresectable stage III patients that do not undergo CRT (34% of patients), RT alone is performed in only 10% of patients [IQR 10–20]. Other approaches include palliative treatment and best supportive care.

Patients continuing treatment after CRT represent 70% of all cases [IQR 50–70]. The mean time from RT completion to patients’ first evaluation is usually less than a month (*n* = 6) or between 1–2 months (*n* = 3), enabling the start of immunotherapy consolidation within 6 weeks (see Table 3). Most physicians (*n* = 8) use a CT scan as a follow-up procedure after RT, and subsequent approaches are often discussed in multidisciplinary team meetings. Surgery following CRT is residual, being considered only in rare situations, especially impacted by patients’ response to CRT.

Additionally, in most countries, the use of Durvamulab is subject to local reimbursement by the National Health System Committee. In the Netherlands, the decision to reimburse the drug within the public health system is made by a specific decision-making forum. In Portugal, an additional local approval is required by the Hospital Pharmaceutical Committee. Physicians from the Netherlands, Norway, Slovenia, and Switzerland reported no current barriers to implementing Durvamulab into daily practice. However, timely evaluation response (Croatia and Israel) and eligibility criteria (Belgium, Greece, Poland, and Portugal) were highlighted as major limiting factors that may hamper ICI use in some nations (see Table 4).

**Figure 1:**
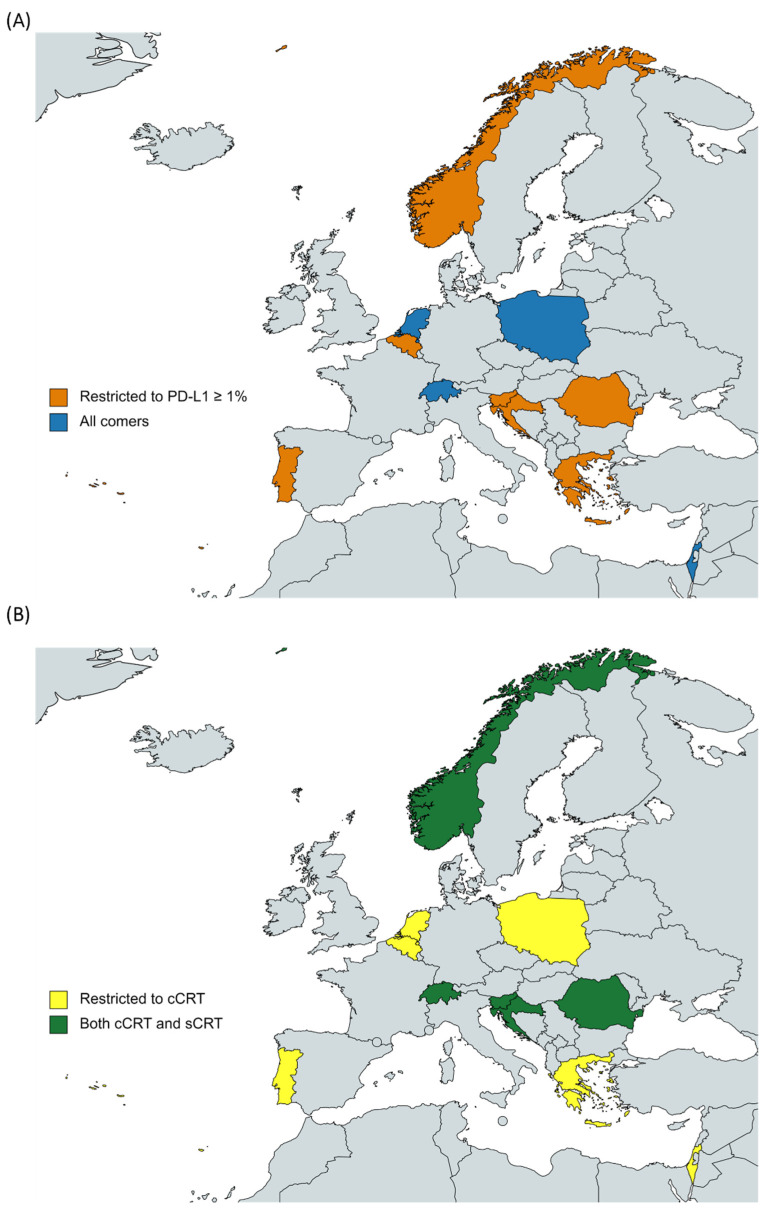
Challenges for Durvamulab implementation in daily clinical practice (**A**) Regulatory criteria for using Durvamulab regarding PD-L1 expression; (**B**) Restrictions of CRT (either concurrent or sequential).

A major controversy between the physicians was whether patients with molecular aberrations should be treated with ICI consolidation. Physicians from Belgium, Greece, the Netherlands, Poland, and Portugal started to treat patients with molecular aberrations (EGFRm/ALK translocation) with Durvamulab after CRT.

Experts agreed that the Durvamulab is well tolerated by the vast majority of patients. The most reported adverse events associated with the therapy are pneumonitis and endocrine events. Oncologists from Greece, the Netherlands and Switzerland also reported skin-related events (e.g., rash), while those from Poland and Romania described gastrointestinal events/colitis. In most countries (*n* = 7/11) adverse events risk management plans during Durvamulab treatment are available (Table 5).

Most physicians (*n* = 10/11) agree that patients should remain on Durvamulab for up to 1 year. Since the PACIFIC study was published quite recently, data are still missing regarding treatment at progression. In case of oligo-progression or isolated brain progression, all experts agreed that local ablative treatment (i.e., surgery or radiosurgery) is the strategy of choice; most of them also consider associated surgery. No consensus on the time to define immune-sensitive disease from completion of Durvamulab until first progression was found among the experts. While physicians from Croatia, Greece, and Romania believe that if a patient progresses 12 months after completion of ICI treatment, he could be considered immune-sensitive, physicians from Israel and Switzerland referred to 6 and 3 months, respectively. Belgium, Norway, Poland, Portugal, The Netherlands, and Slovenia were uncertain about the time to define immune-sensitive disease. Oncologists agreed that treatment following progression depends on progression timing: during or after Durvamulab completion. In most countries (*n* = 7/11, Croatia, Greece, Poland, Romania, Slovenia, Switzerland, and the Netherlands) physicians do not consider treating with ICI if the patient’s disease progresses during Durvamulab treatment. On the other hand, if the progression occurs after Durvamulab completion, reported practices were heterogeneous; most experts mentioned the use of ICI/ICI-based combinations (*n* = 7/11, Belgium, Greece, Israel, Norway, Poland, Romania and Slovenia, Switzerland), but time from Durvamulab treatment completion until new treatment initiation with ICI was not uniform (12 months had passed from the treatment completion: Greece and Romania; 6 months had passed from the treatment completion: Belgium, Norway, Poland; independently of time from treatment completion: Israel, Slovenia and Switzerland) (Table 6).

## 4. Discussion

This study was inspired by the ongoing challenges that physicians face daily in clinical practice when managing a highly complex and multifaceted condition such as stage III NSCLC. Through a multi-national survey in 11 countries with physicians who routinely treat these patients, this study was able to identify their perceptions of the existing barriers to rapid diagnosis, disease staging and treatment selection that may impact clinical and economic outcomes in real-life settings.

While treatment outcomes of stage III NSCLC are improving, the treatment algorithms are still more complex and prolonged, leading to many urgent issues that might affect the ability to adhere to the primary treatment plans. Real-life situations are sometimes different from clinical-trial settings. In clinical trials, patients’ selection criteria are stringent due to their vagueness, ambiguity [26], complexity [27], overly restrictive nature, and lack of patient-centeredness [28]. PACIFIC-R (NCT03798535) is a large international, observational study of patients with unresectable stage III NSCLC who received ≥1 dose of Durvamulab (10 mg/kg Q2W) as part of an AstraZeneca-initiated expanded access program (September 2017–December 2018) that aimed to address these differences in real-life settings [29].

The prompt diagnosis and staging of NSCLC provides vital information on the anatomical details and extent of cancer with respect to the size of the primary tumor, lymph nodal status, and the presence or absence of metastasis in distant organs, which can guide more assertive therapeutic decisions [30,31]. The National Comprehensive Cancer Network and European Society of Medical Oncology recommend an intravenous contrast agent-enhanced chest CT, 18-fluorodeoxyglucose ([18F]-FDG)-PET-CT and, depending on the guidelines, a brain MRI for staging of NSCLC before treatment with curative intent [32,33,34]. MRI was found to be typically used for diagnosis in most countries unless contraindications to this procedure exist. Only in Poland is a PET scan the standard procedure performed on all admitted patients; for those with brain involvement suspicion, a CT scan is conducted.

Confirmation of mediastinal disease (N2) generally precludes NSCLC management [35]. Yet, the role of EBUS for pathological confirmation was not uniform among the interviewed physicians. While in Belgium, Greece, and the Netherlands this procedure is often performed for clarification of PET positive lymph nodes (either to confirm the presence of tumoral cells or negate other pathologies, namely sarcoidosis); in Israel and Poland, EBUS is not commonly conducted when a PET scan is highly positive (high standardized uptake values—SUV). On the other hand, all of the participants agreed that mediastinoscopy plays an important role in stage III patients, especially to confirm clinically relevant nodal disease when EBUS is not able to detect metastasis (e.g., negative or inconclusive results). Mediastinoscopy was also highlighted as a practice for proving downstaging following neoadjuvant treatment [36].

Physicians from Belgium, Israel, Greece, and Romania reported that they usually start ChT at least one cycle before RT due to practical issues that prevent the beginning of RT at day 1. In Poland, concurrent ChT and RT from day 1 is often the selected strategy. In the Netherlands, the use of induction cisplatin-based ChT differs throughout centers across the country. A common scheme, however, is without an induction cycle and consists for example of a CRT scheme with low weekly doses of cisplatin-docetaxel (cisplatin 20 mg/m^2^ and docetaxel 20 mg/m^2^) for 5 weeks. Although the choice of a ChT scheme is based on histology subtype and potential toxicity, ChT protocols vary widely, and an optimal regimen has not been determined. Etoposide-based treatments were commonly mentioned by the studied countries for NSCLC—except by Israel; however, indications and regimens are not uniform. In Greece and Romania, schemes with etoposide are prescribed for most patients, especially to alleviate toxicity. According to the literature, platinum and etoposide-based protocols have favorable outcomes for survival without compromising HRQoL (e.g., acceptable toxicity); commonly used combinations include cisplatin-etoposide and carboplatin-paclitaxel. Patients should be selected not only on the basis of their anticipated response to therapy, but also on how well they are expected to tolerate the therapy [10,11,12,13].

This study revealed that approximately 35% of patients diagnosed with stage III NSCLC in the 11 participating countries do not undergo CRT. According to the physicians, the underlying reasons for this are mostly related to patients’ performance status (e.g., those with poor status can only undergo RT), patients’ age (e.g., older patients usually receive only RT), or comorbidities (e.g., cases of interstitial lung disease or very low diffusing capacity for carbon monoxide can use only ChT). Some physicians also consider surgery after CRT in certain rare cases such as pathological downstaging or PanCoast tumor (up-front decision). According to the experts from Israel, surgery is optional in this scenario, but should not be ignored altogether.

All of the participants agreed that the availability of ICIs (e.g., Durvamulab, pembrolizumab, nivolumab, ipilimumab, atezolizumab) adds multiple options to be considered as consolidation therapy, offering a promising strategy to improve outcomes following cCRT. More recently, substantial clinical evidence has emerged in favor of a synergistic effect between different therapeutic approaches for stage III NSCLC [36]. The antitumor immunogenic effects of radiation can act as an adjuvant to checkpoint blockade. Theoretically, the combination of RT and ICI might lead to enhanced responses by increasing the exposure or altering the presentation of tumor-related antigens to immune system cells [4]. Although current research studies are still investigating the timing and duration of ICI treatments [14,37], all experts agreed that ICI should be started soon after RT completion.

This research found that the median time between the completion of CRT and the initial evaluation was usually less than a month, allowing for initiation of Durvamulab within 6 weeks, which is within the same time window as in the referenced PACIFIC study. This fact is particularly relevant given the exploratory analysis of the PACIFIC trial showing that patients who started treatment with Durvamulab within the first 14 days of RT had improved efficacy compared with those starting therapy 14 days or more following RT completion. Treatment duration is a crucial consideration due to its potential impact on patients’ HRQoL and with respect to the costs. Currently, no correlation has been found between longer treatment duration and increased survival in advanced NSCLC; the optimal duration with immunotherapy is unknown. Experts reported treating patients for one year based on the results of the PACIFIC trial [15].

Additionally, there are still some regulatory barriers and differences in clinical practices among countries (see Figure 1) that limit the use of ICI in real life, especially regarding PD-L1 expression and previous CRT (limited to concurrent CRT or expanded to sequential CRT).

The intention to treat EGFR mutated patients with Durvamulab is not uniform among the international cohort of experts. Physicians from Greece, the Netherlands and Poland stated they do not treat these patients with immunotherapy. In Israel, stage III patients are usually not tested for these mutations and, even if they present a positive result, the common practice is to use only tyrosine kinase inhibitors (TKI) when radical locoregional treatment is not feasible. In Romania and Portugal, on the other hand, patients can be treated with Durvamulab in this scenario, regardless of their genetic profile. In September 2018, the European Medical Agency (EMA) authorized consolidation treatment with Durvamulab only in patients with PD-L1 expression ≥1%, based on an unplanned post-hoc analysis suggesting that using Durvamulab is not beneficial to patients with PD-L1-negative tumors; however, this decision has been highly criticized by the scientific community. Despite this fact, local approval has been granted in Israel, Poland, Switzerland, and the Netherlands regardless of PD-L1 score, showcasing the uniformity across countries represented within this panel. Furthermore, the US Food and Drug Administration (FDA) had already approved Durvamulab as a new standard of care regardless of PD-L1 expression in February 2018 [13,15].

In the US, a single medical center (Veterans Hospital, Birmingham) identified the barriers to consolidative Durvamulab usage by means of a retrospective analysis of health records of veterans with stage III unresectable NSCLC from October 2017 to August 2019 (*n* = 34 patients). The authors found that only 41% of stage III NSCLC patients did CRT and less than one-third of the initial population underwent further treatment with Durvamulab. This was likely due to the difference between clinical trial and real-world patient populations. The most common reasons for not initiating CRT were poor performance status and comorbidities, while the most common reasons for not providing Durvamulab were toxicities during or following CRT [24].

It is known that adverse events of ICI may quell the enthusiasm for using these therapies in settings where patients have already experienced toxicity from CRT [38]. One concern about initiating an anti-PD-1/PD-L1 monoclonal antibody shortly after a definitive dose of radiation is the theoretical combined risk of pneumonitis. That adverse event is a recognized complication of both RT and ICI. However, despite the rate of all-grade pneumonitis being higher with Durvamulab than with placebo (12.6% vs. 7.7%), rates of grades 3–5 pneumonitis are low in both arms, with no meaningful differences (1.9% vs. 1.7%) according to the PACIFIC trial [16,17,18,19]. Additionally, these unique side effects caused by ICI (i.e., immune-mediated adverse events) are commonly manageable with standard treatment algorithms [39].

The access and costs of immunotherapy may also limit its use in practice. Given the high cost of Durvamulab infusion, the survival threshold required to be cost-effective may differ between countries [4]. For the US scenario, a cost-effectiveness study based on the PACIFIC trial showed that Durvamulab consolidation therapy resulted in an additional 1.34 life years (LY) and 1.01 QALY, with a final ICER of $138,920 per QALY vs. placebo. Subgroup analyses demonstrated that Durvamulab was more cost effective for patients with NSCLC, followed by 25% or greater PD-L1 expression (willingness-to-pay threshold of $150,000 per QALY) [40]. Another analysis with respect to the Italian National Health Service perspective showed an ICER of €62,131 per LY and €42,322 per QALY when using Durvamulab vs. placebo for consolidation therapy in stage III NSCLC. Durvamulab was considered cost-effective when a discount of 13% and 30% on its official price was applied, considering all other drugs priced according to official or maximum selling prices, respectively [41].

Upon discontinuation of ICIs, either due to disease progression, adverse events, or even treatment completion, data on treatment options are still limited. Within the PACIFIC trial, an exploratory analysis to characterize the first subsequent following discontinuation of Durvamulab was performed. Overall, among patients who received a subsequent disease-related anticancer treatment, platinum doublet ChT (16.4%) was the most common approach (33.2%) [42]. However, another course of ICIs could be feasible, though the benefit of this approach is still poorly defined. Some authors propose that another round of ICIs should be carefully considered and adjusted based on three different scenarios: treatment after resolution of immune-related toxicity; progression after a completed prior course of ICI; progression during treatment with immunotherapy [43,44].

Other dilemmas and challenges for the integration of immunotherapy in unresectable stage III NSCLC clinical practice still need to be investigated. Durvamulab has not been studied in patients who receive tri-modality therapy, and it is therefore unknown whether patients who undergo resection after cCRT should also be offered ICI as a component of their curative treatment plan. Research to optimize the use of ICI in terms of timing, duration of therapy, and time to define immune-sensitive disease are required. Interventions should be developed to address socioeconomic and system level barriers in order to improve delivery of lung-cancer treatment in the different countries.

Finally, considering the complexity and the prolonged nature of the treatment algorithm due to the use of multi-modality approaches including chemotherapy, radiation, and ICI, and given the important differences among European countries’ health system structure and regulations, we propose the following practical recommendations to minimize any deviations from the primary treatment plan:

1. Patients should be treated within a specialized center with sufficient facilities and resources for diagnosis and disease management, including: imaging and nuclear medicine, pathology and molecular biology, medical and radiation oncology, pulmonology and thoracic surgery.
2. Treatment decisions should be reached after comprehensive, in-depth discussion within this multidisciplinary team; the treatment plan must be shared with the patient.
3. We suggest that each patient have a primary physician, preferably a medical oncologist, to act as a ‘reference’ for all clinical decisions and to be responsible for the patient’s treatment.
4. We encourage the designation of a ‘team coordinator’ (e.g., a nurse) to be responsible for organizing all patients’ appointments and to monitor the patient periodically after treatment.
5. The multidisciplinary team should be familiar with the local country’s regulations and reimbursement issues.
6. During the treatment journey, we encourage professionals to discuss with experts from other disciplines the potential adverse side-effects of the treatment (e.g., pain, gastrointestinal toxicity etc.) and how to manage them. This could contribute to improving patients’ quality of life.

## 5. Conclusions

Our study has some limitations. Non-probabilistic convenience sampling in cross-sectional studies may carry a bias in data collection due to under-representation of subgroups, considering that only one reference physician from each country (mostly European-based) was included (i.e., oncologists working in a reference center were invited to participate in the survey). No oncological society was specifically consulted for this study. However, this was an exploratory opinion exercise; no inferences or extrapolation of the data were conducted, and the answers refer to the individual practices in each center from each country. Therefore, we were able to portray the perception of physicians that routinely manage NSCLC patients and provide initial insights into the current barriers and recommendations to address these challenges in real-world clinical practice, mostly in the EU and in Israel (totaling 11 countries). Yet, further evaluations, including by oncologists from other countries and regional trading blocs (e.g., North America, South America, UK, Asia), should be performed. Although the questionnaire was administered at the end of 2020, which could raise concerns about the impact of COVID-19 on the clinical activities evaluated in this study, all of the questions were retrospective in terms of the routine reality prior to the pandemic.

## Figures and Tables

**Table 1:** Current practices for stage III NSCLC staging.

Variable	Category	Total *n* (%)
Clinical staging		
PET scan	Yes	11 (100.0%)
	No	0 (0.0%)
When PET scan is performed	At diagnosis	7 (63.6%)
	Candidates for surgery or radical CRT	4 (36.4%)
Baseline brain MRI in all patients	Yes	8 (72.7%)
	No	3 (27.3%)
Pathological staging		
EBUS	Yes	11 (100.0%)
	No	0 (0.0%)
When EBUS is performed	At diagnosis	6 (54.6%)
	Mediastinal nodes verification	5 (45.4%)
Mediastinoscopy	Yes	7 (63.6%)
	No	4 (36.4%)
PD-L1 evaluation	Yes	9 (81.8%)
	No	2 (18.2%)
EGFR/ALK evaluation	Yes	7 (63.6%)
	No	4 (36.4%)

Note: ALK: anaplastic large-cell lymphoma kinase; CRT: chemoradiation therapy; EBUS: endobronchial ultrasound bronchoscopy; EGFR: epidermal growth factor receptor; MRI: magnetic resonance imaging; PET: positron emission tomography; PD-L1: programmed death-ligand 1 protein.

**Table 2:** Current practices for stage III NSCLC treatment with chemoradiation.

Variable	Category	Total *n* (%)
Multidisciplinary team treatment decision	Yes	11 (100.0%)
	No	0 (0.0%)
Difference between unresectable IIIA or IIIB	Yes	4 (36.4%)
	No	7 (63.6%)
Neoadjuvant ChT	Yes	8 (72.7%)
	No	3 (27.3%)
When neoadjuvant ChT is used before surgery * ^(a)^	Potentially operable cases	5 (62.5%)
	Bulky mediastinal mass	2 (25.0%)
	Tumor size	1 (12.5%)
	Clinical trials	1 (12.5%)
Definition of cCRT	Simultaneous use of ChT and RT at D1 of cycle 1	3 (27.3%)
	At least 2 cycles of ChT administered during the RT, where induction chemotherapy is allowed	6 (54.5%)
	At least 1cycle of ChT administered during the RT, where induction chemotherapy is allowed	2 (18.2%)
Reason for using induction ChT before CRT *	RT delay	5 (71.4%)
	PS	1 (14.3%)
	Tumor size	1 (14.3%)
Reasons for not receiving CRT * ^(b)^	PS	9 (81.8%)
	Comorbidities	5 (45.5%)
	Access	3 (27.3%)
	Tumor size	1 (9.1%)
	Age	2 (18.2%)
Reasons for not receiving CRT as scheduled *	Adverse events	6 (75.0%)
	PS	2 (25.0%)
Is patients’ age a qualifying factor for cCRT?	Yes	4 (36.4%)
	No	5 (45.5%)
	Sometimes	2 (18.2%)
Is patients’ PS a qualifying factor for cCRT?	Yes	11 (100.0%)
	No	0 (0.0%)
Recommend CRT for patients with stage IIIC	Yes	4 (36.4%)
	Whenever possible	7 (63.6%)
Recommend CRT in molecular aberrations	Yes	11 (100.0%)
	No	0 (0.0%)
RT delay hinders cCRT qualification	Yes	6 (54.6%)
	No	5 (45.4%)
Use RT—IMRT	Yes	11 (100.0%)
	No	0 (0.0%)
Use RT—3D-CRT	Yes	5 (45.4%)
	No	6 (54.6%)
Use platinum-based ChT protocols	Yes	11 (100.0%)
	No	0 (0.0%)
Use etoposide-based ChT protocols	Yes	9 (81.8%)
	No	2(18.2%)
AEs during and after cCRT *	Pneumonitis	8 (72.7%)
	Hematological toxicity	4 (36.4%)
	Esophagitis	4 (36.4%)
Is there an AEs risk management plan during cCRT?	Yes	4 (36.4%)
	No	7 (63.6%)

* Physicians were allowed to select more than one answer (sum of variables’ category may be over 100%). ^(a)^ Total sample *n* = 8 (not reported by Israel, Poland, and Slovenia, given that the answer to “Neoadjuvant ChT” was “No”). ^(b)^ Croatia, Greece, and Switzerland did not identify any reason for not completing CRT as scheduled ChT: chemotherapy; CRT: chemoradiation therapy; cCRT: concurrent CRT; IMRT: intensity-modulated radiation therapy; PS: performance status; RT: radiotherapy. Pneumonitis, hematological toxicity, and esophagitis were described as the most serious adverse events during and after cCRT. However, most clinicians (*n* = 7/11) stated that there is no specific adverse events risk management plan for these cases.

**Table 3:** Current practices for stage III NSCLC treatment on post chemoradiation procedures.

Variable	Category	Total *n* (%)
Timing of first evaluation after RT completion	<1 month	6 (54.5%)
	1–2 months	3 (27.3%)
	1–3 months	1 (9.1%)
	3 months	1 (9.1%)
Follow-up procedures after RT	Repeat PET	1 (9.1%)
	Follow up by CT	8 (72.7%)
	Both PET and CT	2 (18.2%)
Discussion with multidisciplinary team after CRT	Always	3 (27.3%)
	>75% of patients	3 (27.3%)
	<50% of patients	2 (18.1%)
	Never	3 (27.3%)
Is surgery (after CRT) considered?	Yes	1 (9.1%)
	In case of downstaging	3 (27.3%)
	No	7 (63.6%)
Factors influencing surgical decisions *	Response to CRT	6 (85.7%)
	Tumor size/invasion	2 (28.6%)

* Physicians were allowed to select more than one answer (sum of variables’ category may be over 100%) CRT: chemoradiation therapy; RT: radiotherapy. The current challenges in each evaluated country for using the recent ICI Durvamulab during stage III NSCLC is depicted in Figure 1. This therapy is registered and reimbursed in all countries, yet some nations have limiting criteria for its use. In Israel, the Netherlands, Switzerland, and Poland Durvamulab is used regardless of the patient’s PD-L1 score. On the other hand, cCRT (only) is a requirement in Belgium, Greece, Israel, the Netherlands, Poland, and Portugal.

**Table 4:** Approval of Durvamulab and challenges for its implementation into daily clinical practice.

Variables	Belgium	Croatia	Greece	Israel	Norway	Poland	Portugal	Romania	Slovenia	Switzerland	The Netherlands
Durvamulab registered	Yes	Yes	Yes	Yes	Yes	Yes	Yes	Yes	Yes	Yes	Yes
Durvamulab reimbursed	Yes	Yes	Yes	Yes	Yes	Yes	Yes	Yes	Yes	Yes	Yes
Reimbursement date	May 2020	November 2020	2017	January 2019	October 2019	January 2021	October 2019	January 2021	August 2019	2018	April 2019
Durvamulab reimbursement/local approval	--	--	NHSC	NHSC	NHSC	Therapeutic Program	Pharmaceutic. Committee	Different authorities ^(a)^	Prescribing physician	NHSC	Pharmaceutic. Committee
Optimal time to start Durvamulab after CRT	<6 weeks	--	<6 weeks	<2 weeks	<2 weeks	<6 weeks	4–6 weeks	<6 weeks	<2 weeks	<6 weeks	<6 weeks
Barriers to implement Durvamulab in practice	Eligibility criteria ^(b)^	Evaluation response ^(c)^	Eligibility criteria ^(b)^	Evaluation response ^(c)^	None	Eligibility criteria ^(b)^	Price, Eligibility criteria ^(b)^	Adverse events	None	None	None
Treatment with Durvamulab for up to 1 year	No	Yes	Yes	Yes	Yes	Yes	Yes	Yes	Yes	Yes	Yes

^(a)^ Ministry of Health, the National Health Insurance Authority, and the National Drugs and Medical Devices Agency. ^(b)^ Patients’ eligibility criteria for using Durvamulab. ^(c)^ Timely evaluation response. NHSC: National Health System committee.

**Table 5:** Safety and tolerability of Durvamulab in daily clinical practice.

Variables	Belgium	Croatia	Greece	Israel	Norway	Poland	Portugal	Romania	Slovenia	Switzerland	The Netherlands
AEs risk management plan with Durvamulab	Yes	No	Yes	Yes	Yes	Yes	No	No	Yes	No	Yes
Durvamulab main AEs: *Pneumonitis*	Yes	Yes	Yes	--	Yes	Yes	Yes	Yes	Yes	No	Yes
Durvamulab main AEs: *Endocrine events*	Yes	No	Yes	--	Yes	No	Yes	Yes	Yes	Yes	Yes
Durvamulab main AEs: *Others*	--	--	Skin-related	--	--	GI tract	--	Colitis	--	Skin-related	Skin-relatedColitis

AEs: adverse events; GI: gastrointestinal

**Table 6:** Current dilemmas following Durvamulab progression.

Variables	Belgium	Croatia	Greece	Israel	Norway	Poland	Portugal	Romania	Slovenia	Switzerland	Netherlands
Treatment of choice in brain—oligoprogression	LAT Surgery	LAT	LAT	LAT	LAT Surgery	LAT Surgery	LAT Surgery	LAT Surgery	LAT Surgery	LAT	LAT
Treatment of choice if a patient progresses after the completion of 12 months treatment with Durvamulab ^(f)^	ICI/ICI-based combination, if more than 6 months has passed from the treatment completion	Other	ICI/ICI-based combination, if more than 12 months passed from the treatment completion	As per 1st line treatment * independently of time from treatment completion	ICI/ICI-based combination, if more than 6 months has passed from the treatment completion	ICI/ICI-based combination, if more than 6 months has passed from the treatment completion	Other ^(a)^	ICI/ICI-based combination, if more than 12 months passed from the treatment completion	As per 1st line treatment* independently of time from treatment completion	As per 1st line treatment * independently of time from treatment completion	Other ^(b)^
Treatment of choice if a patient progresses during treatment with Durvamulab	As per 1st line treatment * independently of time from treatment completion	ICI would not be considered an option	ICI would not be considered an option	ICI/ICI-based combination, if more than 3 months has passed from the treatment completion	Other ^(c)^	ICI would not be considered an option	Other ^(d)^	ICI would not be considered an option	ICI would not be considered an option ^(d)^	ICI would not be considered an option	ICI would not be considered an option
Time to define immune-sensitive disease ^(e)^	Uncertain	12 months	12 months	6 months	Uncertain	Uncertain	Uncertain	12 months	Uncertain	3 months	uncertain

(a) In Portugal, 1st line treatment is usually selected, depending on the time from treatment completion. (b) The Netherlands performs NGS and PD-L1 assessment, preferably on a fresh tumor sample. and addresses treatment options accordingly. (c) In Norway, ChT+ICI would be considered a treatment option if this approach suits the patient. (d) In Portugal, 1st line treatment is usually selected, independently of time from treatment initiation with Durvamulab. Yet, ChT is the most frequent treatment. (e) In Slovenia, ChT would be considered a treatment option. (f) From Durvamulab completion until first progression. * As per 1st line treatment refers to ChT, ChT+ICI, ICI mono, other. Note: ChT: chemotherapy; ICI: immunotherapy; LAT: local ablative treatment.

## Data Availability

The data presented in this study are available within the manuscript. Further information is available on request from the corresponding author.
